# Medication adherence in older adults with chronic diseases: a scoping review of barriers, facilitators, and effective interventions

**DOI:** 10.3389/fragi.2026.1642652

**Published:** 2026-04-20

**Authors:** Doris Cardona-Arango, Valeria Santacruz-Restrepo, Alejandra Rendón-Montoya, Juliana Madrigal-Cadavid, Alejandra Segura-Cardona, Jorge Iván Estrada-Acevedo

**Affiliations:** 1 Independent Researcher, Medellín, Colombia; 2 Pharmacoepidemiology and Risk Management Research Group, HelPharma, Medellín, Colombia; 3 Faculty of Psychology, CES University, Medellín, Colombia

**Keywords:** adherence interventions (MeSH), aged, assessment of medication adherence, chronic disease, comparative effectiveness research

## Abstract

**Objective:**

Medication adherence in older adults with chronic diseases is a public health challenge, given the growing and irreversible aging of the population, with direct repercussions on clinical outcomes and collective wellbeing. This scope review seeks to identify the main barriers and facilitators of pharmacological adherence and effective evidence-based interventions to optimize it.

**Methods:**

A scope review was conducted for the period 2015–2025. Forty-one observational and interventional scientific studies (clinical trials) were selected from PubMed, Scopus, Web of Science, and ScienceDirect. Initially, a search was performed in six databases covering public health, medicine, life sciences, and biomedicine (PubMed and ScienceDirect), evidence-based healthcare (Cochrane Library), social sciences, arts, and humanities (Scopus and Web of Science), and research output, with an emphasis on Latin America, Spain, and Portugal. The four databases with the largest number of publications on the older adult population were selected, including topics such as medication adherence (compliance with pharmacological treatment and prescribing recommendations), medication persistence (uninterrupted continuity in medication recovery and administration), and patient prioritization interventions using automated mechanisms. The final selection of articles was carried out by three experts, who performed a critical appraisal of the evidence. The discrepancies were resolved by two other researchers, following the identification, screening, selection and inclusion phases of the PRISMA-2020 guidelines. The analysis of the information was carried out through synthesis and narrative integration.

**Results:**

Individual barriers were identified, including demographic (age, sex, educational level, and income), psychological (anxiety, depression, and self-efficacy), cultural (beliefs, fatalism, and stigmas), physical and mental health status (multimorbidity and cognitive impairment), and pharmacological (number of medications and adverse effects) factors. Facilitators identified are related to the health system (continuity of care, fragmentation of care, assertive communication, access, and provision of medicines). The interventions include personalized health education.

**Conclusion:**

Medication adherence in older adults should be addressed with comprehensive and sustainable interventions. These interventions combine pharmacist education, technological support, continuous monitoring, and patient participation in therapeutic decision-making. Strategies should be designed with a collaborative approach involving patients, families, and healthcare professionals, ensuring measurable clinical outcomes and improving their quality of life.

## 1 Introduction

Chronic noncommunicable diseases are health conditions with slow progression, long duration (Goodman et al., 2013; De Dienheim-Barriguete et al., 2020; Solarte et al., 2016). They increase in prevalence with the ageing of the population (Liquori et al., 2022), the growing and irreversible trend of the increasing proportion of older adults in society imposes several additional challenges. Providing comprehensive healthcare to an aging population, responding to more people with chronic diseases, a rational use of the resources of the health system by promoting it, to seek an old age with quality of life, to guarantee timely access to medicines, and to educate in therapeutic adherence, among others. At the individual level, epidemiological factors and risks accumulate with age, leading to chronic diseases, several of them simultaneously (multimorbidity) (Iwakiri et al., 2024), which leads to the daily and constant consumption of one or more medications and leads to polypharmacy (more than five concurrent medications) (Masnoon et al., 2017).

Three out of four people suffer from chronic diseases (75%) (Fong, 2019) and for this reason, the World Health Organization (WHO) recommends establishing timely health interventions. To guarantee prevention, control of progression, and slow down its growth, it becomes necessary to focus on pharmacological, nutritional, medical treatments, and healthy lifestyle habits (Reyes, 2021). Guaranteeing the opportunity for access to health technologies of great relevance, such as medicines, by the health system, and compliance by the patient (Murphy et al., 2022).

In addition to the timely and adequate administration of medications, the continuity of drug therapy administration to patients based on the prescriber’s recommendations has taken on great relevance as the main factor affecting therapeutic effectiveness. Described under different terms over the years, and with some differences in concepts, such as “concordance,” “agreement,” “therapeutic alliance,” “compliance,” and finally, “adherence,” highlight the importance of the patient’s role within drug therapy (Vrijens et al., 2012; Pagès-Puigdemont et al., 2018).

Finally, and in accordance with the taxonomy proposed by Vrijens et al. (2012), “adherence” is recognized as the most widely used concept in the current literature and the one that best describes the current reality, as it recognizes the patient as an active participant in therapy. It is defined as a process by which patients take their medication as prescribed, divided into three quantifiable phases: initiation, implementation, and discontinuation of treatment. This scope review is based on this pase. It is mediated by factors that include: sociodemographic (age, gender, race, level of education, residential area) (Burnier and Egan, 2019); patient (income level, socioeconomic status, economic barriers, beliefs, fears, risk perception, social support, etc.) (Pan American Health Organization, 2023; Basak et al., 2014; Faisal et al., 2022); disease (severity, symptoms, disability, availability of treatments, etc.) (Foley et al., 2023; Al Bawab et al., 2021); treatment (duration, therapeutic failures, adverse events, polypharmacy, medication errors, etc.) (Selvakumar et al., 2023); and health system (access to healthcare and medicines, inadequate infrastructure, lack of resources, lack of professionals, or those without adequate training, among others) (Padilla Vinueza and Morales Solís, 2020; Ortega et al., 2018; Christopher et al., 2023). On the contrary, not complying with treatment increases the number of hospitalizations (Mongkhon et al., 2018), adverse events (Dilla et al., 2009), as well as wear and tear on health personnel (Christopher et al., 2022).

In turn, pharmacological adherence is a key factor for the effectiveness of therapies and improvement in health status. It is particularly critical in patients with chronic pathologies, especially older adults (Laba et al., 2018), where higher rates of non-adherence have been found (Burnier and Egan, 2019; Marcum et al., 2013; Nili et al., 2022; Menditto et al., 2021). Since they are the ones who suffer from the most chronic pathologies for prolonged periods and with related multimorbidity (Faisal et al., 2022), in addition to presenting greater difficulties in accessing medicines (Foley et al., 2023).

Access to medicines by this age group is a cause for concern for the countries of the Americas (Pan American Health Organization, 2025), with multiple barriers for obtaining their medications in an affordable and timely manner. Violating their rights to equality and non-discrimination (United Nations Human Rights, 2025), typifying a type of violence against the elderly or abuse by negligence, understood as the denial of medicines, food, or access to medical care in institutions (Yon et al., 2019).

Given the relevance of this problem, various strategies have been proposed to improve therapeutic adherence. Educational interventions, caregiver support, simplification of pharmacological regimens, and the use of digital technologies for medication monitoring and reminders are some of them (Nieuwlaat et al., 2014; Akhter et al., 2022). However, the effectiveness of these strategies continues to be an area of active research, since adherence is a multifactorial phenomenon that requires a comprehensive and individualized approach for adequate optimization. Wilhelmsen and Eriksson (2019) in their narrative review of systematic reviews that evaluated the effectiveness of different interventions in patients in order to improve adherence rates concludes that although a positive effect of these interventions has been demonstrated on the clinical results of the population, and on adherence; to date no strategy has been able to impact all the environments and factors that affect it.

In this context, pharmacological adherence in older adults with chronic diseases is a challenge for public health, given the growing and irreversible aging of the population, with direct repercussions on clinical outcomes and collective wellbeing. This scope review seeks to identify the main barriers and facilitators of medication adherence and effective evidence-based interventions to optimize it.

## 2 Materials and methods

A literature review was conducted between January and February 2025, following the guidelines established in the PRISMA-2020 statement (Preferred Reporting Items for Systematic Reviews and Meta-Analyses) (Page et al., 2021). The search focused on observational studies and interventions, with both quantitative and qualitative approaches, published between 2015 and March 2025, in English and Spanish, that addressed medication adherence and persistence with treatments used in the management of chronic diseases, effective interventions for compliance with healthcare professionals’ instructions, and the causes of discontinuation of pharmacological therapies. For this process, the Rayyan software (https://new.rayyan.ai/- artificial intelligence platform) was used where information from the different databases was entered and according to the inclusion and exclusion criteria.

The following PICoS question was designed for the review:

P: Chronically ill older adults
I: adherence and/or persistence to prescribed medications
Co: intervention strategies for non-adherent patients
S: Medication Adherence/Persistence Studies of Older People

### 2.1 Identification

Initially, six databases were selected from the following areas: public health, medicine, life sciences, and biomedicine (PubMed and ScienceDirect); evidence-based healthcare (Cochrane Library); social sciences, arts, and humanities (Scopus and Web of Science); and research focused on Latin America, Spain, and Portugal. The four databases with the largest number of publications on medication adherence in the older adult population were selected: PubMed, Scopus, Web of Science, and ScienceDirect.

### 2.2 Information sources and search strategy

Table 1 details the search terms used for the articles, according to the database consulted and the filters applied during the selection process. Boolean operators (OR, AND) were used to combine the terms: “medication adherence,” “pharmacological adherence,” “therapeutic persistence,” and “chronic diseases.” In addition, a manual search was conducted in the reference lists of the included studies to identify relevant literature not retrieved in the initial search. This strategy yielded 1211 articles, with 144 duplicates Table 1.

**TABLE 1:** Information sources and search strategy.

Articles identified by database	Search terms	Included articles
PubMed (108) (MeSH + field tags)	(“Medication Adherence”[Mesh] OR “Medication Compliance”[Mesh] OR adherence[tiab] OR “medication adherence”[tiab] OR “therapeutic persistence”[tiab] OR “treatment fidelity”[tiab]) AND (“Chronic Disease”[Mesh] OR “chronic disease”[tiab]) AND (“Aged”[Mesh] OR aged[tiab] OR “older adult”[tiab] OR elderly[tiab]) Filters: Publication date: 01/01/2015–31/03/2025; Languages: English, Spanish; People Search performed: January 20 to February 15, 2025	18
Scopus (436) (TITLE-ABS-KEY)	TITLE-ABS-KEY (“medication adherence” OR adherence OR “therapeutic persistence” OR “treatment fidelity”) AND TITLE-ABS-KEY (“chronic disease”) AND TITLE-ABS-KEY (aged OR “older adult” OR elderly) AND (PUBYEAR >2014 AND PUBYEAR <2026) Languages: English, Spanish Search performed: January 20 to February 15, 2025	7
Web of Science (Yang et al., 2023) Core Collection (TS field)	TS=(“medication adherence” OR adherence OR “therapeutic persistence” OR “treatment fidelity”) AND TS=(“chronic disease”) AND TS=(aged OR “older adult” OR elderly) Timespan: 2015–2025; Languages: English, Spanish; Document Types: Article, Review Search performed: January 20 to February 15, 2025	5
ScienceDirect (577) (Advanced search)	(“medication adherence” OR adherence OR “therapeutic persistence” OR “treatment fidelity”) AND “chronic disease” AND (aged OR “older adult” OR elderly) Years: 2015-2025; Subject area: Medicine/Health Sciences; Article/Review Search performed: January 20 to February 15, 2025	11
Total 1,211		41

### 2.3 Protocol registration and amendments

It is worth clarifying that the terms “catastrophic illness” and “high-cost illnesses” were included at the beginning of the literature search, but were removed during the article selection phase, since older adults often suffer from multiple chronic illnesses simultaneously, and it is the interaction between different therapeutic regimens that can lead to medication discontinuation, which is the focus of this review.

### 2.4 Screening

In this phase, a preliminary evaluation of titles and abstracts was conducted to exclude studies unrelated to the objective of this review, such as research addressing chronic diseases like hypertension and diabetes mellitus but not addressing adherence to prescribed treatments for these diseases. These actions resulted in the exclusion of 863 articles, and 204 proceeded to the next phase.

### 2.5 Operational definitions

This review considered the taxonomy of adherence, such as that presented by Vrijens et al. (2012), and measurements of medication adherence (Morisky Medication Adherence Scale, pill count, and validated self-report, among others).

### 2.6 Inclusion and exclusion criteria

Studies conducted in older adults (aged 60 and over) between 2015 and 2025 (March) were included, regardless of their place of residence (home or long-term care facility). Studies related to adherence to medications for chronic diseases were considered. Interventions designed to improve adherence based on digital technologies, automated reminders, and multidisciplinary support strategies were also included. Studies in populations with primary psychiatric disorders were excluded, given the potential interference of these conditions with adherence to prescribed medical treatments. Letters to the editor, case studies, and studies without full-text access were also excluded Table 2.

**TABLE 2:** Inclusion and exclusion criteria.

Inclusion criteria	Exclusion criteria
- Studies published: 2015-2025 (March) - Languages: English and Spanish - Participants: seniors (60 ≥años ) - Areas of expertise: medicine, geriatrics, pharmacology - Events: Chronic diseases - They use medications for these chronic events - Observational and interventional studies, with a quantitative and qualitative approach	- Studies with preliminary results - Non-human Studies - Populations with primary psychiatric disorders - Letter to de editor - Case reports

### 2.7 Study selection and reviewer agreement

**FIGURE 1:**
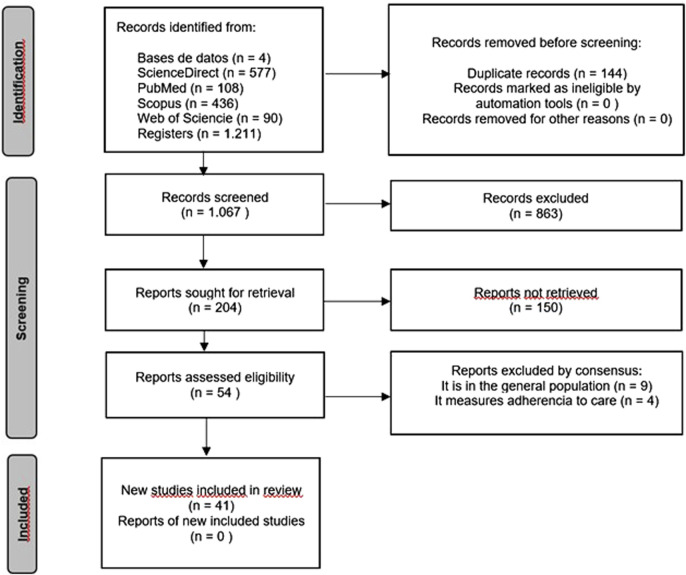
Flowchart of the process of identification, screening, eligibility, and inclusion of studies according to the PRISMA statement.

Titles/abstracts and full texts were selected by two independent reviewers; in case of disagreements, these were resolved by consensus or through arbitration by a third reviewer. Any discrepancies were resolved by a third investigator, resulting in the consensus withdrawal of 13 studies, as they were deemed not to contribute to the objective of this review (conducted in the general population (n = 9), measures adherence to care (n = 4). This left 41 articles ultimately included in the review Figure 1.

### 2.8 Evaluation of methodological quality

The quality of the selected studies was assessed using validated tools. For observational studies (cohorts and case-controls), the Strobe guideline (STrengthening the Reporting of OBservational studies in Epidemiology) was used (von Elm et al., 2008). It allows quality to be assessed in terms of selection, comparability and results. For intervention studies (clinical trials), the Cochrane Risk of Bias (RoB 2) tool was applied (Nejadghaderi et al., 2024). It assesses the risk of bias in key aspects such as randomization, allocation concealment, and blinding of participants and evaluators.

### 2.9 Data extraction

The extracted data were recorded in an Excel spreadsheet, which included the following variables: authors, year, country, study design, sample size, identified barriers and facilitators, and finally, details of the interventions implemented to improve medication adherence in older adults.

### 2.10 Analysis and synthesis of results

The data analysis was performed using synthesis and narrative integration, focusing on medication adherence, barriers, facilitators, and interventions implemented to prevent treatment discontinuation.

### 2.11 Ethics committee

It is noted that the scope review was not presented to any ethics committee, older adults were no longer surveyed, and the moral rights of the authors of the studies were respected.

## 3 Results

41 articles were selected that met all the requirements for analysis in the scope review (Leung et al., 2015; Viana et al., 2015; Hegde et al., 2016; Jamerson et al., 2016; Yap et al., 2016; Neoh et al., 2017; Lau et al., 2017; Lee et al., 2018; Pradhan and Panda, 2018; Zhang et al., 2018; Lai et al., 2018; Avazeh et al., 2019; Hanna et al., 2020; Polat et al., 2020; Qiao et al., 2020; Di Novi et al., 2020; Liu et al., 2020; Arain et al., 2021; Manjavong et al., 2021; Conde-Caballero et al., 2021; Chu et al., 2021; Kim et al., 2022; Yu et al., 2022; Hamrahian et al., 2022; Koohestani and Baghcheghi, 2022; Zhao-rong et al., 2022; Feda et al., 2022; El-Sayed et al., 2023; Skerry et al., 2023; Fadil et al., 2023; Jeyalakshmi et al., 2023; Dagnew et al., 2024; Wu et al., 2024; Chen et al., 2024; Lee and Choi, 2024; Carandang et al., 2024; Odubanjo et al., 2024; Smith-Ray et al., 2024; Wen et al., 2024; Lim and Woo, 2025; Dou et al., 2025). With a complete reading, qualitative and quantitative data were extracted, according to the proposed related objective of obtaining the factors, barriers, facilitators, and interventions of pharmacological adherence. Manuscripts selected were published in different countries and continents. It is striking that the number of articles increases over time, evidencing an interest in delving into the issue of pharmacological adherence, now that an increase in the elderly population in health services is observed, multi-pathological and polymedicated, but with greater awareness of their state of health and the contribution of self-care in their improvement Table 3.

**TABLE 3:** Studies included in the review.

Authors		Year	Country	Sample size	Design	Journal
1.	Leung et al. (2015)	2015	China	3,167	Observational	Geriatr Gerontol Int
2.	Viana et al. (2015)	2015	Brazil	27,333	Observational	Revista de Saude Publica
3.	Hegde et al. (2016)	2016	India	184	Observational	Geriatr Gerontol Int. 2016 Dec;
4.	Jamerson et al. (2016)	2016	United States	4,136	Observational	J Am Geriatr Soc.
5.	Yap et al. (2016)	2016	​	​	Review	J Clin Gerontology and Geriatrics
6.	Neoh et al. (2017)	2017	Malaysia	79	Observational	Geriatr Gerontol Int.
7.	Lau et al. (2017)	2017	China	593	Observational	J Public Health.
8.	Lee et al. (2018)	2018	United States	4,818	Observational	Prev Chronic Dis
9.	Pradhan and Panda (2018)	2018	India	425	Observational	Biomedical and Pharmacology Journal
10.	Zhang et al. (2018)	2018	China	311	Observational	Patient Preference and Adherence
11.	Lai et al. (2018)	2018	China	258	Observational	BMC Geriatrics
12.	Avazeh et al. (2019)	2019	Iran	222	Observational	Jundishapur Journal of Chronic Disease Care
13.	Hanna et al. (2020)	2020	Israel	22	Qualitative	Int J Nurs Stud
14.	Polat et al. (2020)	2020	Turkey	316	Observational	European Journal of Geriatrics and Gerontology
15.	Qiao et al. (2020)	2020	China	780	Observational	Patient Education and Counseling
16.	Di Novi et al. (2020)	2020	Italy	40,658	Quantitative	Health Economics (United Kingdom)
17.	Liu et al. (2020)	2020	Singapore	323	Observational	Patient Preference and Adherence
18.	Arain et al. (2021)	2021	Canada	91	Intervention	BMC Geriatr
19.	Manjavong et al. (2021)	2021	Thailand	250	Observational	Int J Gerontology
20.	Conde-Caballero et al. (2021)	2021	Spain	34	Qualitative	Journal of Personalized Medicine
21.	Chu et al. (2021)	2021	China	300	Observational	Geriatr Nurs.
22.	Kim et al. (2022)	2022	United States	54,500	Observational	Explor Res Clin Soc Pharm
23.	Yu et al. (2022)	2022	China	272	Observational	Geriatric Nursing
24.	Hamrahian et al. (2022)	2022	​	​	Review	Patient Preference and Adherence
25.	Koohestani and Baghcheghi (2022)	2022	Iran	358	Observational	Iranian Journal of Ageing
26.	Zhao-rong et al. (2022)	2022	China	7,088	Observational	Chin J Public Health
27.	Feda et al. (2022)	2022	Saudi Arabia	114	Observational	J Pharmaceutical Negative Results
28.	El-Sayed et al. (2023)	2023	Egypt	200	Observational	Geriatric Nursing
29.	Skerry et al. (2023)	2023	Canada	57	Mix	Canadian Pharmacists Journal
30.	Fadil et al. (2023)	2023	Saudi Arabia	501	Observational	International Journal of Clinical Practice
31.	Jeyalakshmi et al. (2023)	2023	India	360	Observational	Patient Preference and Adherence
32.	Dagnew et al. (2024)	2024	Ethiopia	336	Observational	Front Pharmacol
33.	Wu et al. (2024)	2024	China	622	Observational	Explor Res Clin Soc Pharm
34.	Chen et al. (2024)	2024	China	1,515	Observational	Journal of Medical Internet Research
35.	Lee and Choi (2024)	2024	South Korea	​	Observational	Geriatric Nursing
36.	Carandang et al. (2024)	2024	Philippines	24	Qualitative	Explor Res Clin Soc Pharm
37.	Odubanjo et al. (2024)	2024	South Africa	130	Observational	South African Family Practice
38.	Smith-Ray et al. (2024)	2024	United States	317,613	Intervention	J Manag Care Spec Pharm
39.	Wen et al. (2024)	2024	China	523	Intervention	J Sichuan University (Medical Sciences),
40.	Lim and Woo (2025)	2025	South Korea	8,656	Observational	Geriatric Nursing
41.	Dou et al. (2025)	2025	China	963	Observational	Medical Science Monitor

### 3.1 Pharmacological adherence of older adults

Low pharmacological adherence in older adults represents a challenge due to the significant clinical and economic implications. It is estimated that between 20% and 55% do not follow medical indications regarding the use of their drugs, which compromises the effectiveness of treatment and control of chronic diseases (Polat et al., 2020; Fadil et al., 2023). In multimorbid geriatric patients, non-adherence is a determining factor in the progression of their conditions, increased risk of exacerbations and clinical complications. This translates into an increase in emergency department consultations, hospitalizations, hospital readmissions, overload on health systems and mortality (El-Sayed et al., 2023).

Population aging is associated with a high prevalence of multimorbidity and polypharmacy, which further complicates pharmacological adherence. In Turkey, it was reported that more than 80% of people over 60 years of age suffer from at least one chronic disease, while approximately 23% have four or more simultaneous conditions, which translates into the need for multiple drugs for their management, increases the probability of therapeutic errors and abandonment of treatment (Polat et al., 2020).

From an economic perspective, non-adherence has a considerable financial impact, both for health systems and for families and caregivers. It has been documented that an improvement in adherence could significantly reduce the medical costs associated with managing preventable complications (Lee et al., 2018). In many cases, it is family members who assume the consequences of inadequate management of chronic diseases in the elderly, facing an increase in care needs due to adverse events such as strokes, heart attacks or the progressive deterioration of heart function, events that could be avoided with adequate compliance with pharmacological treatment.

In summary, pharmacological adherence in older adults with chronic diseases is a complex phenomenon, determined by multiple factors, with profound implications for individual health and the lack of therapeutic compliance perpetuates an adverse cycle of uncontrolled disease, the appearance of complications and an intensive use of health resources (Hanna et al., 2020).

### 3.2 Barriers and facilitators of pharmacological adherence in older adults

Several authors agree that therapeutic adherence in older adults is a multifactorial and complex phenomenon (Neoh et al., 2017). Recent literature identifies a wide range of factors that influence pharmacological adherence in older adults. Ranging from individual patient characteristics to elements of the health system, they can act as barriers or facilitators of therapeutic compliance, classified as follows:

- Demographic barriers. Non-modifiable demographic conditions (age and sex) increase the probability of pharmacological non-adherence if they are added to economic conditions such as poverty. Older age has varying effects: some studies found better adherence in those over 85 years of age (Lim and Woo, 2025), while in Chinese patients with heart disease aged 65–74 years, they reported more barriers (Yu et al., 2022). Female sex has been associated with lower adherence in certain contexts (for example, women with hypertension or diabetes tend to be less compliant with treatment than men) (Wu et al., 2024).

Other demographic conditions can be modified, such as educational level and income. In Korean study, individuals with a lower educational level showed greater adherence, possibly due to less skepticism toward medical instructions. Low health literacy often makes it difficult to follow treatments (Lee and Choi, 2024). Contrary to that, in an Iranian study, a high educational level presents a more satisfactory adherence to medication (Avazeh et al., 2019).

Economic constraints impose a tangible barrier due to difficulties in affording medicines, resulting in rationing or abandonment of treatment. In the United States, it was observed that older adults without subsidies and with a high annual deductible (-US$500) were significantly less likely to be adherents (AOR 0.48) (Kim et al., 2022). Likewise, several demographic barriers such as sex, education, income, marital status and living alone can be combined (Feda et al., 2022).

- Psychological barriers. Psychological factors such as anxiety, depression, self-efficacy, and hopeless disease affect therapeutic behavior.

An Egyptian study in 200 older adults showed that higher levels of anxiety about illness and fatalistic beliefs correlated with low pharmacological adherence (r = −0.16 and −0.19, respectively) (El-Sayed et al., 2023). Likewise, depression and feelings of hopelessness reduce the motivation to take medications. For example, depressed patients often have negative attitudes towards their medications, requiring specific emotional support interventions (Chen et al., 2024). Self-efficacy–the patient’s low confidence in the management of their disease–was associated with lower adherence to treatment, in two studies carried out in China with more than seven thousand participants (Zhao-rong et al., 2022; Wu et al., 2024).

- Cultural barriers. Understanding and respecting cultural factors is key, as culturally sensitive interventions have proven to be more successful in promoting adherence. The barriers encountered have to do with shared beliefs, cultural fatalism, and social stigmas about some diseases.

A set of shared beliefs determines whether or not a patient accepts a treatment (Qiao et al., 2020; Liu et al., 2020; Chu et al., 2021). Some Iranian and Korean older adult populations show a preference for traditional herbal remedies (Koohestani and Baghcheghi, 2022) Instead of Western drugs, which can affect adherence (Lee and Choi, 2024). Culture profoundly influences attitudes toward medications, as some seniors consider medications to be toxic (Odubanjo et al., 2024) or harm your liver, kidney, and/or gastrointestinal health.

Cultural fatalism–the belief that the disease has an inexorable and predetermined course–tends to take root culturally and can lead to resignation, reducing the effort to follow treatments (El-Sayed et al., 2023). In addition, cultural taboos or stigmas about certain diseases (e.g., depression) may prevent older adults from taking their medications regularly or recognizing the need for them (Chen et al., 2024).

- Physical and mental health status barriers. The presence of multiple pathologies (multimorbidity) and physical frailty is determinant of pharmacological adherence.

The number of conditions and multimorbidity is a barrier to adherence to the treatment of a given disease, since each one has its own medication regimen, care, and increases the therapeutic burden. In a study carried out in South Korea in 2025, it was documented that patients with a high comorbidity index (e.g., Charlson ≥3) are at greater risk of not adhering to complete treatment (Lim and Woo, 2025). Likewise, Dou et al. (Dou et al., 2025) found non-adherence statistically associated with physical frailty and social frailty.

The simultaneous management of chronic diseases (diabetes, hypertension, dyslipidemia, etc.) generates stress and anxiety, due to drug-disease interactions, which further complicates the situation (Pradhan and Panda, 2018). In addition to the above, the severity of concomitant chronic diseases, having more chronic or longer-lasting diseases (Kim et al., 2022) was identified as a barrier to adherence (Yu et al., 2022).

Mentally, memory disorders and dementia can be a barrier to pharmacological adherence. Age-related cognitive decline makes it difficult to comply with therapy due to memory problems or confusion, they may forget the prescribed doses or take them twice. Dementia (e.g., early-stage Alzheimer’s disease) has been identified as one of the strongest predictors of non-adherence in the elderly (Lim and Woo, 2025) and mild cognitive impairment in older patients can affect the ability to handle multiple drugs and follow complex instructions (Jamerson et al., 2016; Polat et al., 2020).

- Pharmacological barriers. Polypharmacy involves a delicate balance between effectively treating diseases and not overwhelming the patient with impractical regimens. This is why the use of multiple drugs and their side effects are factors related to pharmacological adherence in older adults with chronic diseases.

Regimens that involve taking many tablets a day, or at different times, lead to mistakes and omissions. The use of multiple drugs (Dagnew et al., 2024), between 23% and 39% of older adults consume five or more medications simultaneously, and the complexity of the regimen, frequent doses, and multiple daily doses are critical factors that decrease adherence (Zhang et al., 2018; Lai et al., 2018; Polat et al., 2020). Likewise, a prolonged treatment (Hamrahian et al., 2022; Lee and Choi, 2024) or potentially inappropriate medication is a predictor for non-adherence (Pradhan and Panda, 2018).

Medication side effects or adverse events from taking multiple medications (dizziness, nausea, confusion, etc.) may cause the patient to discontinue one of the prescriptions on their own. In a study conducted in Thailand, it was found that not experiencing side effects was linked to better adherence, suggesting that minimizing adverse reactions (e.g., by adjusting doses or simplifying therapy) can improve adherence (Manjavong et al., 2021).

- Barriers of the Health System. The availability of medicines is crucial for good pharmacological adherence. Shortages, multiple administrative procedures in the management of drugs, or fragmentation of care between different professionals, can interrupt treatment even in motivated patients. Continuity of care, support from nursing professionals, pharmaceutical programs, and assertive communication are factors that improve adherence in older adults.

The interruption of healthcare due to abrupt transitions (for example, from hospital to home) makes it difficult to follow treatments, has been associated with abandonment and confusion in older patients, reducing their adherence. In contrast, when the system provides close accompaniment, such as home care programs with nursing staff visiting the patient, pharmacotherapeutic follow-up, and health education by pharmaceutical professionals, adherence improves markedly (Lim and Woo, 2025). The fragmentation of care or consultations with multiple uncoordinated specialists or poorly integrated information increases the burden on the patient. Some authors describe the management of their diseases as “a lonely battle” when they do not feel supported or receive clear guidance from the system (Skerry et al., 2023).

The support of nursing professionals and pharmacists is associated with pharmacological adherence. A Korean observational study of older adults with diabetes found that those who received care through specialized community nurses had higher adherence rates than those without this support (Lim and Woo, 2025). The participation of pharmaceutical professionals and programs reduces the cost of care for patients with diabetes, hypertension, and hyperlipidemia and is associated with better adherence (Smith-Ray et al., 2024). In Wisconsin, the proportion of low-income seniors with optimal adherence to their drugs increased to 80% in 5 years, thanks to a State program (Kim et al., 2022).

Factors related to the health system play a central role, since poor or hasty communication by health professionals can leave the patient with doubts or little motivation to follow the indicated plan (Lau et al., 2017; Skerry et al., 2023). Therefore, honest, symmetrical, and assertive communication between patients and professionals generates confidence in older adults and avoids self-medication, the use of alternative medicinal plants (Koohestani and Baghcheghi, 2022), and the modification of doses. In addition to providing knowledge and accompaniment in treatments for chronic diseases, for life (Hegde et al., 2016). In written communications, the labels of medicines should also be reviewed, as there is no distinction between generic and brand names (Neoh et al., 2017).

Lack of access to medicines remains a problem in settings with fragile health systems. For example, in Brazil, 14% of older adults could not obtain any of their prescription medications, a figure that rose to 22% among those with four or more diseases (Viana et al., 2015). The distance to pharmaceutical institutions is also a geographical barrier (Di Novi et al., 2020) evidenced in a population-based study carried out in Italy, which increases mortality and increases the use of other services of the health system.

Without adequate social support, the elderly may prioritize one condition over another, giving up on following so many indications. Therefore, older patients with multimorbidity require additional support strategies (caregiver supervision, special packaging, visual or sound reminders) to ensure the correct intake of their medications (Leung et al., 2015). Family support was also found to be associated with medication adherence in adult patients with hypertension and diabetes, living in rural areas of Karnataka, India (Jeyalakshmi et al., 2023).

Evidence suggests that family support favors the internalization of healthy habits and the maintenance of long-term treatment. In a qualitative study of patients who had suffered a myocardial infarction, participants identified their wives and family members as key elements in their recovery and adherence. In contrast, social isolation, absence of a support system, and overprotection are significant barriers to maintaining the therapeutic regimen (Hanna et al., 2020).

### 3.3 Effective interventions to improve drug adherence

Pharmacological adherence in older adults is a phenomenon influenced by a complex network of interrelated factors that become barriers and facilitators for therapeutic compliance. The scientific literature indicates that non-adherence cannot be attributed to a single cause, but responds to the interaction of multiple demographic, physical, psychological, social, and structural barriers within the health system, which must be addressed in a comprehensive manner (Hanna et al., 2020). In this sense, interventions to improve therapeutic adherence are designed with a multidimensional approach that includes personalized education by pharmaceutical professionals, the incorporation of automated technologies and healthcare models.

#### 3.3.1 Educational interventions led by pharmaceutical professionals

The promotion of therapeutic adherence in older adults has gained special relevance due to their frequent and direct contact with pharmaceutical professionals. They facilitate and eliminate barriers that affect the continuity of the therapeutic regimen, with strategies such as proactive clinical follow-up, pharmacovigilance, structured medication review, individualized counseling, pharmacotherapeutic interventions, and decision-making in conjunction with other health professionals (Smith-Ray et al., 2024). In this sense, the active incorporation of the pharmacist into the interdisciplinary care team for the elderly, either through home pharmaceutical care services or in the field of community pharmacies, constitutes an evidence-based strategy to optimize pharmacological adherence in this population (Smith-Ray et al., 2024).

There is evidence that pharmacists who are well-positioned in the community to support older people with self-management of treatments, information about the disease, side effects, drug interaction, polypharmacy, and continuity of care improve medication adherence (Skerry et al., 2023). It is recognized that cognitive impairment, functional limitations, and multimorbidity of the elderly hinder pharmacological adherence. That is why a technological solution such as telepharmacy emerges, which overcomes access barriers, geographical distances, and reduces risks to patients and the health system. Its shortcoming is the limited technological competence of adults and the economic limitations to have cell phones and access to the internet, and the design of intuitive platforms that allow them to communicate effectively (Carandang et al., 2024).

#### 3.3.2 Use of technology (mHealth) and electronic reminders

Technological innovations have opened up new opportunities to strengthen therapeutic adherence in older adults, providing tools that facilitate the monitoring of medical treatments, such as digital voice assistants. In Spain, a digital assistant called *Assistant on Care and Health Offline* (ACHO) was designed and evaluated, conceived to operate without an internet connection and adapted to older adults living in rural areas with limited technological access. This device allows you to send automated voice and text reminders for taking medications, daily consumption records, telephone alarms to family members and caregivers if there are forgetfulness in the taking of a medication (Conde-Caballero et al., 2021). There are also software based on artificial intelligence (AI) such as real-time, voice or text *chatbots* that interact with older adults about their medications and give them reminders and instructions to improve medication adherence (Dou et al., 2025).

#### 3.3.3 Electronic medication delivery system

Recently, the use of medication dispensing systems (MDS) through electronic machines that distribute medicines has increased. Showing an decrease in healthcare visits and self-perceived physical and mental health (Arain et al., 2021).

#### 3.3.4 Patient portals and remote monitoring

The use of digital technologies in the field of health has made it possible to develop innovative tools to improve therapeutic adherence in older adults with chronic diseases. Among these strategies, *Patient Portal Systems* have gained relevance as an effective resource for remote monitoring and disease self-management. These portals provide patients and their caregivers with access to key information about their treatment, including personalized care plans, medication records, educational materials, and direct communication channels with healthcare professionals. Its implementation has been shown to facilitate continuous monitoring and encourage greater responsibility in therapeutic compliance. For example, a study with 18 months of follow-up, carried out in 523 Chinese older adults diagnosed with coronary heart disease, this strategy showed 83.3% treatment compliance, notably higher than the 70% recorded in the control group with traditional care (Wen et al., 2024).

#### 3.3.5 Online communities and peer support

The psychosocial component of therapeutic adherence strengthens the creation of spaces where patients share experiences, resolve concerns, and receive support from people in similar situations (v.gr.: social network *PatientsLikeMe* - https://www.patientslikeme.com).

Virtual patient communities, specialized forums, discussion groups on social networks, and interactive platforms have proven to be valuable tools to improve the perception of treatment, promote adherence. They become a space where patients can exchange practical advice, concerns, and disease management. In a study focused on patients with major depression, it was shown that participation in online forums dedicated to this pathology had a positive impact on adherence to antidepressants, an effect mediated by a greater perception of social support and a revaluation of treatment (Wen et al., 2024). In that study, it was identified that both educational content provided by health institutions and positive testimonials from other users played a key role in the motivation to continue treatment.

#### 3.3.6 Home care

Home care, especially for older adults with reduced mobility or multiple barriers to access medical care, is based on scheduled visits from health professionals, such as doctors, nurses, pharmacists, and therapists. They not only monitor vital signs and adjust medication but also provide health education in the patient’s everyday context, facilitating the integration of the treatment into their daily lives. A study conducted in South Korea looked at therapeutic adherence in older adults with diabetes mellitus who received home healthcare (HHC) compared to those who relied exclusively on outpatient consultations. The findings found that the presence of a greater number of nurses specialized in HHC was associated with a higher probability of medication adherence (Lim and Woo, 2025).

## 4 Discussion

With the progressive aging of the population, more and more elderly people have multiple chronic pathologies that require complex pharmacological regimens, which increases the risk of non-adherence and its clinical and economic consequences (Polat et al., 2020). Creating challenges, with a demographic phenomenon, the sustainability of healthcare systems, and the improvement of the health status and quality of life of the populations (Yap et al., 2016). The evidence reviewed also supports that optimal treatment adherence is associated with lower mortality in older adults with chronic diseases, compared to those who have poor adherence to their treatment (El-Sayed et al., 2023). These findings underscore the need to address pharmacological adherence from a comprehensive perspective, with multidimensional interventions that involve patient, family, health system, and technologies and innovations guided by artificial intelligence.

Studies have shown that optimizing therapeutic adherence can have a more significant impact on the health of the population than improving medical treatments themselves, since the effectiveness of a pharmacological intervention depends to a large extent on adequate compliance by the patient (Gudala et al., 2022). However, adherence tends to decline with age, with cognitive decline, functional barriers, lack of social support, and increasingly complex therapeutic regimens (Smaje et al., 2018). This is consistent with the findings of this review, where age (Lim and Woo, 2025), social support (Jeyalakshmi et al., 2023), and physical (Dou et al., 2025) and mental health status (Jamerson et al., 2016; Lim and Woo, 2025) were barriers for pharmacological adherence.

It is estimated that, in developed countries, only 50% of patients with chronic diseases follow their long-term treatments correctly, while in low and middle-income countries this proportion is even lower, reflecting asymmetry in access to information, health resources, and continuity of care (Hanna et al., 2020; Polat et al., 2020). In older adults, adherence is less than 43%, resulting from a lack of standardized instruments for their measurement (self-report, tablet counting, event monitoring systems, electronic devices, pharmacy records, among others) (Jeon et al., 2022).

The need to improve pharmacological adherence has motivated the creation of telemonitoring systems. Many of them are expensive and difficult to use for older adults, who do not have access to the internet and smartphones (Carandang et al., 2024), or the sensory and cognitive ability to follow indications for one or more simultaneous treatments (polypharmacy) (Giardini et al., 2016). This motivated the creation of QR code-based systems attached to the packaging of medicines delivered to patients (Capranzano et al., 2021).

Likewise, Kanyongo et al. (2025) published the results of using seven machine learning algorithms to classify patients with non-communicable diseases (diabetes and/or hypertension) and predict adherence. For the classification, the following algorithms were used: *deep neural network* (DNN), *support vector machines* (SVM), *decision tree* (DT), *logistic regression* (LR), *Naïve Bayes* (NB), *K-Nearest Neighbour* (k-NN), and *random forest* (RF). RF can record the minimum errors in patient classification and suggest follow-up strategies. These authors recommend developing learning systems for patient prioritization that improve adherence, disease management, and healthcare.

The World Health Organization divides adherence measurements into subjective (medical assessment and self-report) and objective (electronic devices, ingestible biosensors, pill counting, secondary use of databases, among others). Compiled in a review published in 2024 by Gackowski et al. (2024), that provides seven conclusions related to the ten methods studied: there is no standard method; subjective methods depend on the disease; electronic devices are accepted by patients; objective measurements (ingestible biosensors) are very accurate and reliable but do not show the reasons for non-compliance; the combination of both measurements is required in the measurement of adherence, and finally, they mention the use of intelligence (AI), artificial platforms and machine learning in the creation of algorithms that monitor the patient in a personalized way, but they recognize that they are expensive.

The incorporation of artificial intelligence in healthcare has been increasing in recent years (Bohr and Memarzadeh, 2020). It allows for the assessment and prediction of a disease, success of treatments, management of side effects and personalized assistance of management plans, and the design of algorithms that use large datasets that capture genomic profiles and contributing environmental factors to aid in the development of precise therapies (Lavertu et al., 2018). Even with all these advances, more AI use is required among pharmaceutical professionals to identify inappropriate orders and medications and improve medication adherence (Jessica et al., 2025). It can be used for reporting the effectiveness of technological applications on the use of electronic pill bottles or boxes, ingestible sensors, electronic drug management systems, blister technology, patient self-report technology, video-based technology, and motion sensor technology, among others (Mason et al., 2022). Applying it to the elderly population, given the physical and mental limitations, the chronic diseases that many of them suffer, whose non-adherence increases the costs to the health system (Yang et al., 2023).

The authors acknowledge the limitations encountered in preparing this manuscript: few studies with several long-term chronic diseases simultaneously, as this compromises medication adherence and access to treatment in the long term; the method for measuring medication adherence is not standardized; the cost of the identified barriers is not quantified; few studies originate from low-income countries; the literature is dominated by observational studies and has fewer studies on effective interventions and facilitators that improve the health and wellbeing of older adults.

## 5 Conclusion

Pharmacological adherence in older adults with chronic diseases is a multifactorial phenomenon influenced by the following factors: demographic (age, sex, educational level, and income), psychological (anxiety, depression, and self-efficacy), cultural (beliefs, fatalism, and stigmas), physical and mental health status (multimorbidity and cognitive impairment), pharmacological (number of medications and adverse effects), and health system (continuity of care, fragmentation of care, assertive communication, and access to and provision of medicines). The analysis of more than forty studies published between 2015 and 2025 identifies that non-adherence in this population represents a significant clinical challenge, contributing to adverse health outcomes. In addition, it is evident that there is no single effective strategy to improve therapeutic compliance, but that a comprehensive approach that encompasses both the patient and their environment and the healthcare system is required.

Among the main factors associated with adherence identified in literature, cognitive impairment and the burden associated with the presence of multiple diseases and treatments emerge as critical barriers. In contrast, the presence of a strong family and social support network, as well as fluid and effective communication with health professionals, stand out as key facilitators. It has also been observed that emotional factors, such as anxiety about the disease or negative beliefs about the efficacy of medications, can significantly reduce adherence if they are not addressed properly. In turn, economic difficulties are a major limitation for many older adults in the acquisition of their treatments.

In terms of strategies to improve adherence, the literature reviewed supports the implementation of multidisciplinary interventions that combine objective and subjective approaches. Among them with greater empirical support are personalized health education, family accompaniment, the intervention of pharmacists in therapeutic follow-up, the use of technological tools (electronic reminders, digital platforms for patients, prioritization algorithms using artificial intelligence and learning environments, etc.), home care programs, and, in general, person-centered approaches. In particular, the combination of strategies, such as individual education with the simplification of the therapeutic regimen and remote follow-up, has been shown to be highly effective in improving adherence rates, especially in patients with complex medication schemes, polypharmacy, and simultaneous multimorbidity.

In conclusion, optimizing pharmacological adherence in older adults with chronic diseases requires a systematic and personalized evaluation of the individual barriers faced by each patient, along with the implementation of specific measures that reinforce the facilitating factors. It is imperative that health professionals take a proactive role in identifying difficulties in adherence and apply support strategies adapted to each case, from education and reorganization of treatment to family inclusion and the use of technologies assisted by artificial intelligence (machine learning algorithms and big data). In addition, strategies to improve adherence must be sustainable over time, since, in the context of chronic diseases, this behavior must be maintained throughout life. To this end, it is essential to strengthen health systems through policies that favor continuity of care, accessibility to medicines, and the integration of multidisciplinary teams. Only through a solid supportive environment and a patient-centered approach will it be possible to successfully face the challenge of therapeutic adherence in old age, thus contributing to improving the quality of life and health outcomes of this growing population.

Ultimately, medication adherence in older adults should not be seen solely as an individual patient responsibility, but as a public health challenge that requires a coordinated and sustainable approach. The implementation of objective and subjective interventions will reduce avoidable medical complications and hospitalizations. In the face of the progressive ageing of the world’s population, it is necessary to continue researching and innovating in interventions that promote optimal adherence, ensuring that older adults receive the necessary support to manage their treatments effectively and maintain their wellbeing over time.
